# Influence of Implant Geometry on the Surface Strain Behavior of Peri‐Implant Bone: A 3D Analysis

**DOI:** 10.1111/cid.70003

**Published:** 2025-02-07

**Authors:** Moritz Löhlein, Constantin Motel, Manfred Wichmann, Ragai Edward Matta

**Affiliations:** ^1^ Dental Clinic 2, Department of Prosthodontics Universitätsklinikum Erlangen Erlangen Germany

**Keywords:** dental implants, implant biomechanics, implant geometry, implant loading, optical measurement, peri‐implant stress distribution

## Abstract

To ensure long‐term implant success, it is crucial to understand the force transmission from the implant to the surrounding bone. In dentistry, bioengineering methods are applied to investigate these processes. The aim of this study was to analyze the influence of different implant geometries on the surface strain behavior of porcine mandibles under load using a 3D optical camera system in combination with digital image correlation. Four different implant types were subjected to a force of 200 N in three different loading directions (axial, non‐axial 15°, and non‐axial 30°). Under axial loading, parallel‐walled implants exhibited lower surface strain values on the peri‐implant bone compared with tapered implants. However, when subjected to non‐axial loading, these parallel‐walled implants showed a substantial relative increase in strain by approximately a factor of 2.96 compared with axial conditions. At a 30° non‐axial angle, long, tapered implants with a smaller diameter (BLX 3.75) produced lower peri‐implant bone strains than implants with larger diameters and shorter lengths, while short, tapered implants (BLT) demonstrated a lower relative increase in strain (factor ~1.49) from axial to non‐axial loading. Under non‐axial loading, long, tapered implants with a small diameter resulted in lower strains in the peri‐implant bone compared with implants with a larger diameter and shorter length. It was found that non‐axial loads lead to higher strains than axial loads. Therefore, the success of implantation could be significantly influenced by selecting an appropriate implant geometry and the correct angulation of the implant.

## 1 Introduction

Since the concept of osseointegration was introduced over 60 years ago, implantology has developed into a successful and predictable treatment method for replacing missing teeth [1, 2, 3]. Despite this proven rehabilitation method, it is necessary to evaluate the reasons for failure. This includes examining the biomechanical processes and the force distribution from implants to the surrounding bone, as excessive loads can reduce the success of the implantation [4, 5, 6].

According to Esposito et al. [7], various reasons for implant failure are discussed. One cause mentioned is mechanical failure, which includes fractures of the implants and the associated superstructure, possibly involving screws. Iatrogenic failures encompass issues such as incorrect angulation and injury to adjacent anatomical structures. Another important aspect is biological failure, which can be further divided into early and late failures. Early failure occurs when osseointegration is not achieved, indicating a disturbance in the initial healing process. Late failure, on the other hand, refers to the inability to maintain the achieved osseointegration [7, 8, 9].

To achieve osseointegration, several conditions must be met: a biocompatible material, an appropriate macro design and surface of the implant, correct surgical technique, and proper loading of the implant [10, 11, 12]. Osseointegration is influenced by biomechanical stimuli that directly impact the contact between bone and implant. The areas of the implant in direct contact with surrounding bone are responsible for load transmission. The design of dental implants pursues two objectives: first, promoting correct primary stabilization, as osseointegration relies on adequate mechanical stability and a favorable biological environment. Second, facilitating proper load transfer to the surrounding tissues of the implant once secondary or biological stability has been achieved post‐osseointegration [10, 13, 14].

Implants primarily anchored in trabecular bone present a greater biomechanical challenge. This is due to the limited contact between bone and implant caused by the higher porosity of trabecular bone (40%–95%) compared with cortical bone (5%–15%). As a result, there is low primary stability, which can lead to micro‐movements and ultimately promote implant failure. Since bone quality cannot be altered, selecting an appropriate implant design is crucial to minimize stress on the bone‐implant interface in regions with low primary stability and promote osseointegration [15, 16, 17, 18].

The field of biomechanics enables a better understanding of biological systems by analyzing the relationship between the structures involved and their mechanical function. This involves using direct measurements or mathematical models to comprehend the mechanical properties [19, 20].

In osseointegrated implants, occlusal loads are transmitted directly to the surrounding bone without movements facilitated by the natural tooth‐supporting apparatus [21, 22, 23, 24, 25]. In this context, the geometry of the implant influences the distribution of stress and load in the surrounding bone [26, 27, 28, 29]. Higher stresses and strains in the peri‐implant bone can increase the risk of bone damage or marginal bone resorption [30, 31, 32, 33].

The aim of this study was to analyze the influence of various implant geometries on the strain behavior at the surface of porcine mandibles under loads applied with a force machine, using digital image correlation (DIC). The study investigated how parameters such as length, diameter, and taper of the implant affect the superficial strain behavior of the bone under different loading directions.

While stress and strain distribution in peri‐implant bone has been extensively studied using finite element methods, experimental investigations employing DIC remain scarce. This study utilizes DIC as a novel approach to directly measure strain distribution, providing experimental validation and complementing existing computational models. Although various aspects of implant success have been explored, the specific influence of design parameters such as length, diameter, and taper on strain distribution in peri‐implant bone under different loading conditions remains insufficiently understood. Addressing this question is crucial for optimizing implant designs and improving clinical outcomes.

Building on the existing knowledge of biomechanical processes, this study aims to bridge this gap by systematically analyzing the strain behavior of porcine mandibles subjected to varying implant geometries and loading directions.

The null hypothesis of this study posits that the selected implant design parameters (length, diameter, and taper) exert no significant influence on the distribution and magnitude of strain within the surrounding bone. By systematically examining these variables, the aim is to determine whether, and to what extent, they shape the mechanical load conditions and thus the long‐term stability of the implant.

## 2 Material and Methods

In the present study, the research team used pig mandibles obtained directly from a slaughterhouse (Contifleisch GmbH and Unifleisch GmbH, Erlangen, Germany). All specimens were sourced on the same day from animals of similar age and physiological condition, ensuring a uniform tissue quality among the samples. Since the mandibles were freshly harvested as part of the regular food production process, their tissue condition closely resembled that of viable bone. Due to their classification as slaughter by‐products, no ethical approval was required to use these specific samples.

For the experimental setup, the posterior aspect of the ascending ramus of the mandible was chosen due to its comparable dimensions to the human mandible. Rectangular bone segments were cut out using an oscillating saw, and the same anatomical region was consistently selected to ensure standardized dimensions and homogeneity of the bone blocks. The prepared segments were then embedded in a plaster matrix (Fujirock, Type 4 super hard plaster) to facilitate accurate and reproducible testing conditions.

The implant beds were prepared according to the drilling protocol of Straumann GmbH [34, 35].

**FIGURE 1:**
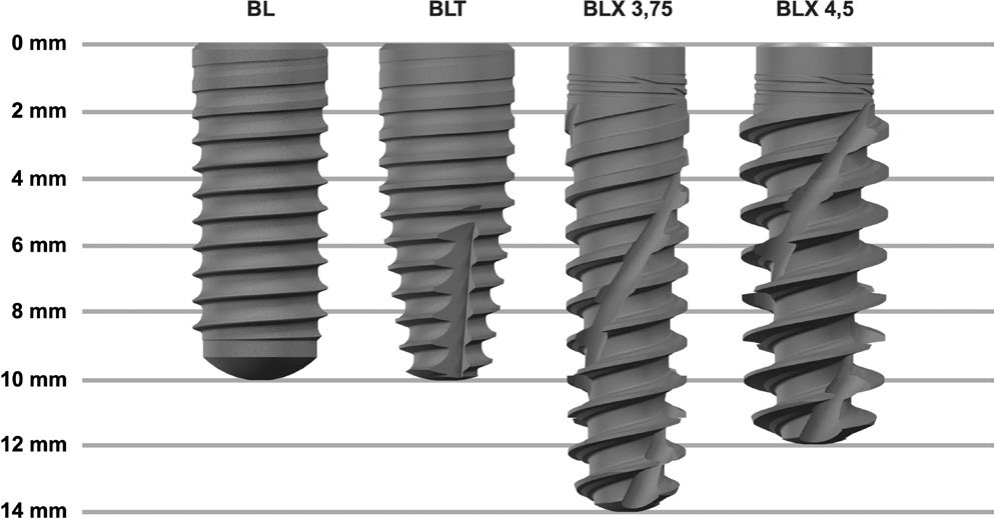
Implant geometries of the four different implants used in the study (images courtesy of Straumann GmbH, Freiburg, Germany).

The following four implant types were used, as shown in Figure 1: BL (diameter: 4.1 mm, length: 10 mm, parallel‐walled), BLT (diameter: 4.1 mm, length: 10 mm, tapered), BLX (diameter: 3.75 mm, length: 14 mm, tapered), and BLX (diameter: 4.5 mm, length: 12 mm, tapered).

Five implants of each type were inserted. The 20 implants were evenly distributed across 8 rectangular porcine bone segments, with either 2 or 3 implants placed in each segment. The dimensions of the bone segments were approximately 8 cm in width and 6 cm in height. Specific areas of the porcine mandible were selected where at least 1.5 mm of bone remained on each side after implant insertion.

For the measurements conducted in this study, the ARAMIS 3D optical camera system (Carl Zeiss GOM Metrology GmbH, Braunschweig, Germany) was used. This system required a contrast pattern on the surface of the test objects (stochastic pattern). To create this pattern, the bone surface was first primed with a white acrylic resin‐based paint (Sparvar spray paint, Spray‐Color GmbH, Merzenich) and then sprayed with a graphite paint (CRC Industries Deutschland GmbH, Iffezheim), resulting in randomly distributed small black dots on the bone surface.

**FIGURE 2:**
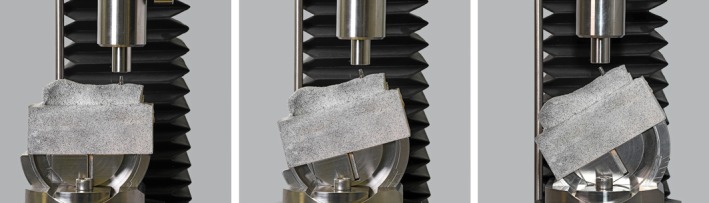
Experimental setup featuring the porcine jaw fixed within an angular plate. A stochastic pattern is observable on the bone's surface. The images depict the three loading directions: Left, axial loading (0°); center, non‐axial loading (15°); and right, non‐axial loading (30°).

Subsequently, the implants were inserted into the pig mandibles and fitted with abutments. Using a compression testing machine (Inspect mini, Hegewald & Peschke Mess‐ und Prüftechnik GmbH, Nossen, Germany), each implant was loaded with 200 N under three different conditions: axial (0°) and non‐axial (15° and 30°). The load application was performed by gradually increasing the force until 200 N was reached, at which point the force level was held steady and strain measurements were recorded. For the angular settings, an angular plate featuring precise reference markings was employed. By aligning the implant's longitudinal axis with the 0°, 15°, or 30° indicators on the plate, the load direction could be accurately and reproducibly established. The distal direction was assigned to the right side of the image in Figure 2.

To measure the strain on the surface of the bone resulting from the load applied by the compression testing machine, the ARAMIS 3D optical camera system (Carl Zeiss GOM Metrology GmbH, Braunschweig, Germany) was used. This is a non‐contact, optical, three‐dimensional deformation measurement system capable of analyzing movements and deformations through DIC [36]. The strain calculation is performed by matching similar gray value distributions in the deformed image to the gray value distributions in the undeformed reference image [37].

The ARAMIS 3D camera was calibrated before each measurement using the calibration plate CP 40/MV 60 mm (Carl Zeiss GOM Metrology GmbH, Braunschweig, Germany) to achieve maximum imaging accuracy. This calibration process involves positioning the calibration plate at predefined distances and orientations within the measurement volume. The system's software identifies reference points on the plate and calculates both intrinsic and extrinsic camera parameters, correcting for lens distortions, perspective errors, and scale factors. By accurately determining these parameters, the 2D image data can be reliably translated into precise 3D coordinates and strain measurements. This reduces systematic errors and ensures that the recorded strain values genuinely represent the specimen's deformation rather than artifacts introduced by the optical system.

The ARAMIS Professional Software (Carl Zeiss GOM Metrology GmbH, Braunschweig, Germany) was used for the inspection and measurement of the test specimens. In this study, the *X*‐axis was defined as the mesio‐distal orientation, with the left side considered mesial (see Figure 1). The *Y*‐axis was defined as the corono‐apical axis, where higher values represented coronal areas and lower values represented apical areas. To differentiate the strain distribution on the bone surface, the measured area was divided into 12 equally sized rectangles. The size of the rectangles was based on the length and diameter of the implants. Thus, the mesio‐distal width of the rectangles for each implant corresponded to the diameter of the implant, and the corono‐apical length of a rectangle corresponded to one‐third of the implant length. Rectangles b, e, and h represented the projection of the implant on the bone surface. Measurement area k was located apically to the implant along its longitudinal axis. Measurement areas a, d, g, j were mesial to the implant, and areas c, f, i, l were distal to the implant. In the study, the principal strain was determined as technical strain, and the strain values were averaged over the fields. For statistical analysis, the mean values were descriptively presented for the four implant types, separated by loading direction, and compared between implant types using one‐way analysis of variance (ANOVA). Additionally, the mean normalized displacement per field for each implant type at each loading direction was graphically represented. For each individual implant, the field with the highest displacement was assigned a value of 1, and the lowest displacement was assigned a value of 0. All other fields received a proportional, normalized value between 0 and 1. These values were then averaged over the five implants of the same implant type. As a result, the displayed values are all below 1. A field would only have received a value of 1 if all five implants had the maximum strain values in the same field. A color legend provided a visual impression of the areas with the highest strain, averaged over the five implants per type. This allowed the strain distribution to be graphically compared among the four different implant types under the three different loading directions. The numbers in the fields and the color legend therefore did not refer to absolute strain values but to the averaged, normalized values of the same implant type over five implants.

**FIGURE 3:**
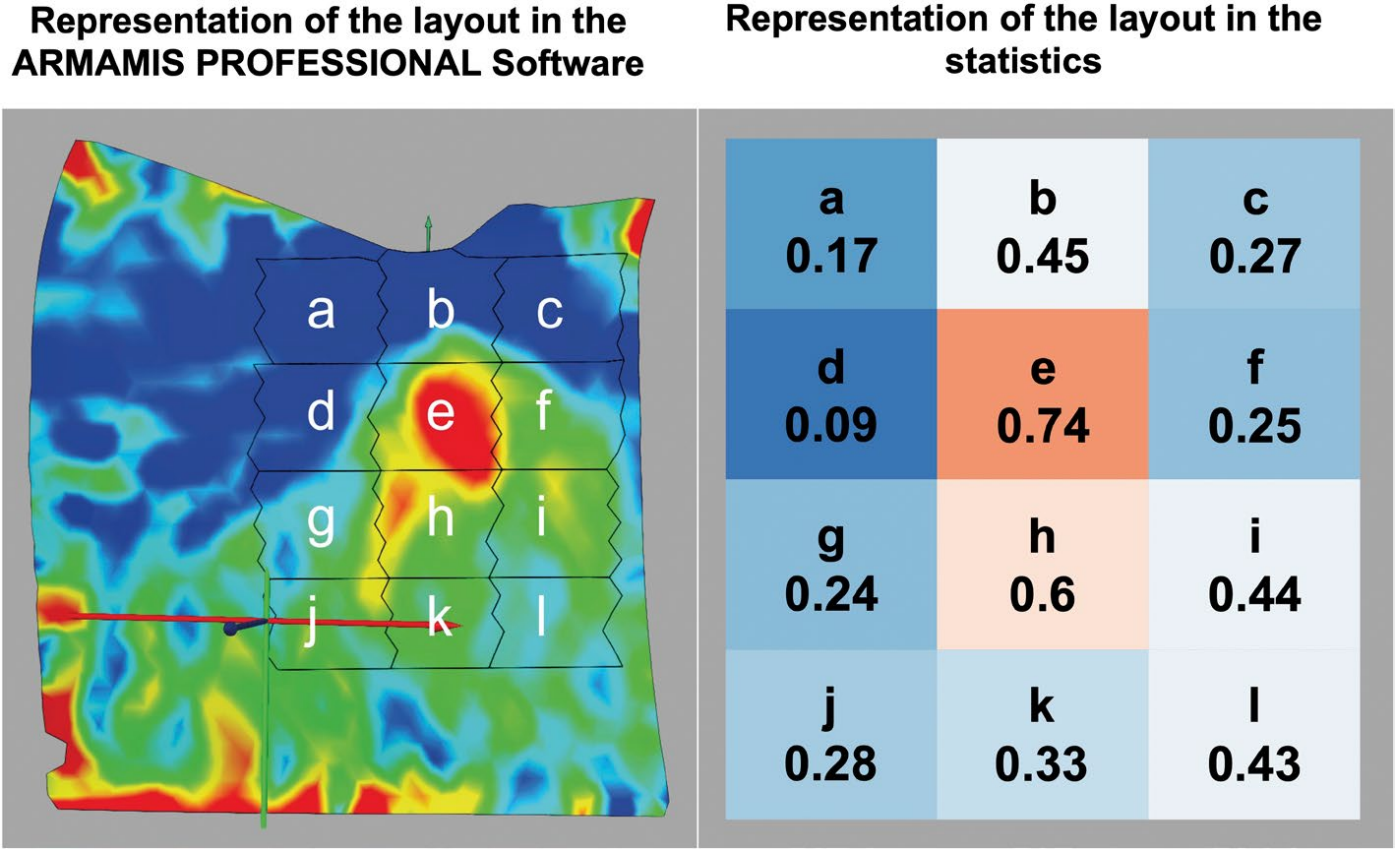
Left: Visualization of surface deformation in the ARAMIS PROFESSIONAL Software as a heatmap (Main change of shape); measurement areas b, e, and h represent the projection of the implant onto the bone surface. Area k is located apically to the implant along its longitudinal axis. Areas a, d, g, and j are mesial to the implant, while areas c, f, i, and l are distal to it. Right: Representation of the layout in the statistics, showing the normalized mean main change of shape on the surface of the bone, averaged over five implants.

Figure 3 (left) shows the representation of the surface strain in the ARAMIS PROFESSIONAL Software as a heatmap. The 12 areas, labeled with letters a to l, are visible and were constructed to differentiate the strain on the bone surface. Figure 3 (right) visualizes the normalized strain distribution on the bone surface. The color and field division provide an impression of the distribution of the strain values.

## 3 Results

When comparing the strain on the surface of the peri‐implant bone under an axial load of 200 N, the mean principal strain for the BL implant was 139.92 μm/m, for the BLT implant was 323.98 μm/m, for the BLX 3.75 implant was 178.96 μm/m, and for the BLX 4.5 implant was 170.62 μm/m. These initial axial loading results, as summarized in Table 1, highlight notable differences in strain values, with the BL implant exhibiting the lowest values.

**TABLE 1:** Averaged maximum strain values (in μm/m) for the four different types of implants, under axial (0°), non‐axial (15° and 30°) loading conditions with a 200 N load.

	Axial load (0°)	Fold	Non‐axial load (15°)	Fold	Non‐axial load (30°)	Overall fold
BL	139.92	1.67	232.99	1.78	414.00	2.96
BLT	323.98	0.99	322.29	1.50	484.08	1.49
BLX 3.75	178.96	1.14	203.72	1.60	325.1	1.82
BLX 4.5	170.62	0.97	166.3	2.08	345.35	2.02

*Note:* Additionally, the table includes the fold change indicating the difference in maximum averaged strain between different experimental conditions and the overall change.

**FIGURE 4:**
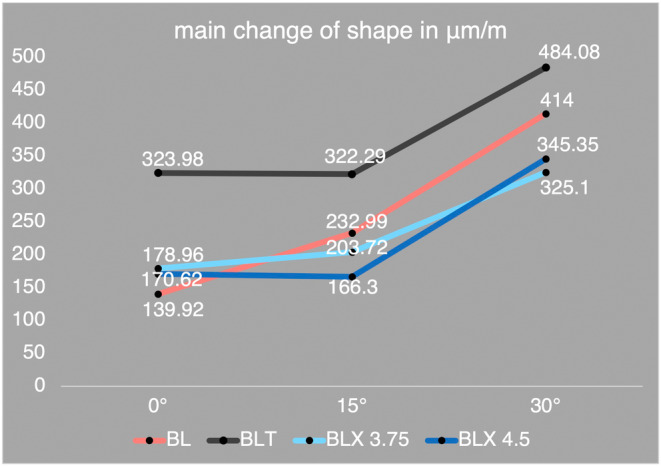
Averaged maximum strain values (in μm/m) for the four different types of implants, under axial‐0°, non‐axial (15° and 30°) loading conditions with a 200 N load.

**FIGURE 5:**
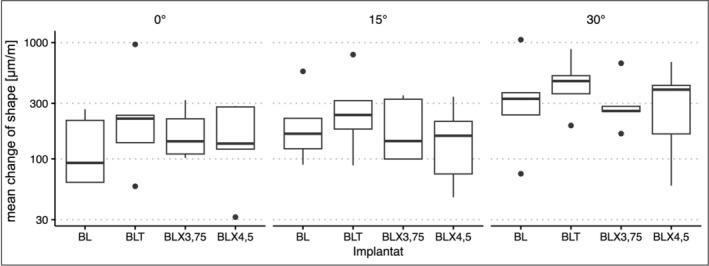
Box‐Whisker plots of the mean maximum strain values in the four different implant types with axial (0°) and the two non‐axial (15° and 30°) force applications.

In comparison, under the “Test type 15°,” where the force was applied at an angle of 15° to the implant's longitudinal axis, there was no or very slight increase in the mean principal strain for the BLT, BLX 3.75, and BLX 4.5 implants. The mean values were 322.29 μm/m for the BLT implant, 203.72 μm/m for the BLX 3.75 implant, and 166.30 μm/m for the BLX 4.5 implant. Only the BL implant showed an increase in mean principal strain (by a factor of 1.67) to a value of 232.99 μm/m when the force direction was changed to 15° distally. Figure 4 (line graphs) and Figure 5 (box‐whisker plots) visually demonstrate this pattern, indicating that the BL implant responds differently to non‐axial loading at 15° compared with the other implant designs.

Under “Test type 30°,” where the implants were loaded non‐axially at an angle of 30° to the implant's longitudinal axis, the mean principal strains increased for all implants. The values were 414.00 μm/m for the BL implant, 484.08 μm/m for the BLT implant, 325.1 μm/m for the BLX 3.75 implant, and 345.35 μm/m for the BLX 4.5 implant. The strain values for the BLX 4.5 implant increased the most (by a factor of 2.08) compared with “Test type 15°,” followed by the BL implant (by a factor of 1.78), the BLX 3.75 implant (by a factor of 1.60), and the BLT implant (by a factor of 1.50). As illustrated in Figure 4 and further detailed in Table 1, these increases underscore the sensitivity of different implant geometries to steeper non‐axial loading angles.

Observing the increase in mean strain values for the different implants when comparing “Test type 0°” to “Test type 30°,” the decreasing order of factors was as follows: 2.96 for the BL implant, 2.02 for the BLX 4.5 implant, 1.82 for the BLX 3.75 implant, and 1.49 for the BLT implant. Figure 5's box‐whisker plots help visualize these relative changes, showing that the BL implant experiences the most pronounced increase under 30° loading.

Figure 4 shows the strain values in μm/m on the *Y*‐axis and the three different loading angles (axial 0°, non‐axial 15°, and non‐axial 30°) on the *X*‐axis. The lines represent the strain distribution of the four different implants (BL, BLT, BLX 3.75, and BLX 4.5) for the respective loading directions.

Table 1 provides a detailed overview of the average maximum strain values for the four different implant types under the three different loading conditions. It also offers insights into the increase in strain values between the different loading directions.

The Box‐Whisker plots in Figure 5 provide a visual overview of the averaged maximum strain values for the four different implant types under axial and the two non‐axial loads.

**FIGURE 6:**
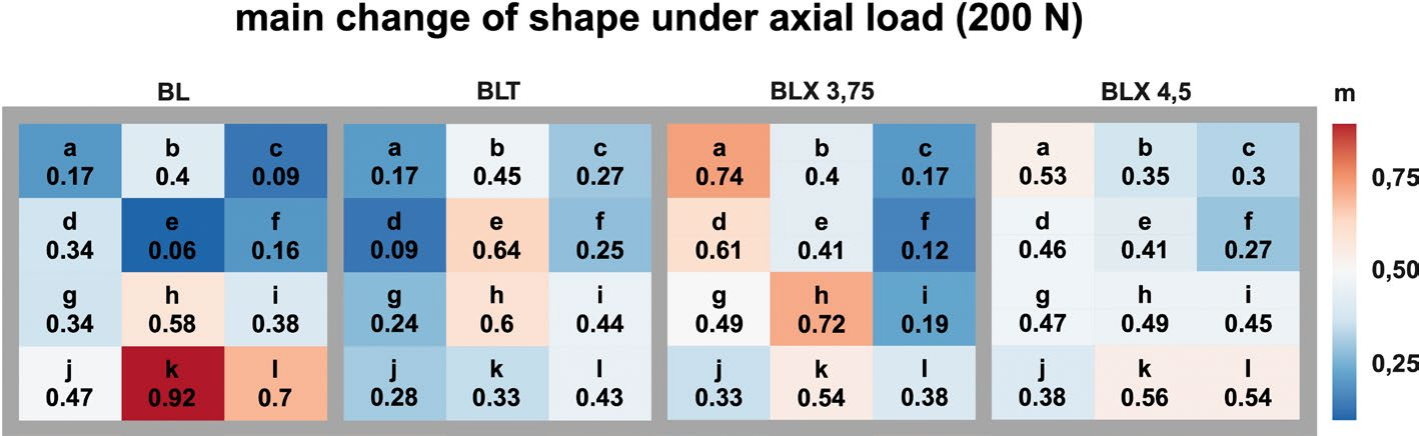
Illustration of the normalized mean change in bone surface shape resulting from axial loading (0°) across four different implant types, each subjected to a 200 N load.

**FIGURE 7:**
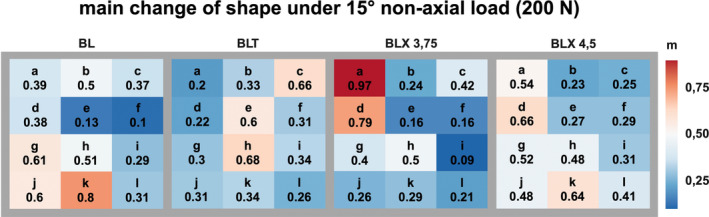
Illustration of the normalized mean change in bone surface shape resulting from non‐axial loading (15°) across four different implant types, each subjected to a 200 N load.

**FIGURE 8:**
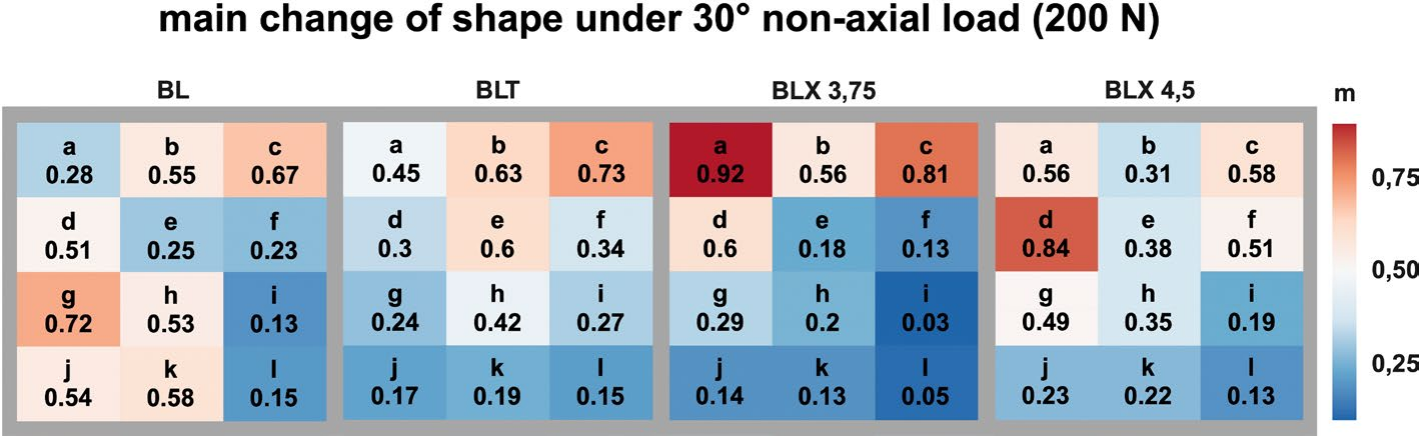
Illustration of the normalized mean change in bone surface shape resulting from non‐axial loading (30°) across four different implant types, each subjected to a 200 N load.

Figures 6, 7, 8 visually represent the strain distribution through averaged normalized fields.

Under an axial load of 200 N, the highest averaged normalized strain values for the BL implant were located in the axial projection caudal to the implant apex. For the BLT implant, the maximum averaged normalized strain values appeared along the implant's longitudinal axis in the middle and caudal thirds of the implant body.

For the BLX 3.75 implant, the highest strain values were observed mesial to the implant body in the crestal region and in the caudal third of the implant body. For the BLX 4.5 implant, the highest strain values were measured apical to the implant apex. As depicted in Figure 6, these spatial patterns emphasize how implant geometry influences the distribution of strain under purely axial conditions.

Under a 15° angled load originating from the distal direction with 200 N, the highest averaged normalized strain values for the BL implant were also located apically to the implant apex. Additionally, increased strain values were observed in the mesial‐caudal region, opposite to the side from which the force was applied.

For the BLT implant, the maximum strain values were found in the middle and apical thirds of the implant body, as well as distal to the implant body in the crestal region.

In the case of the BLX 3.75 implant, the maximum strain values were observed mesial to the implant body in the crestal and middle regions relative to the implant body.

With the BLX 4.5 implant, maximum strain values were measured mesial, centrally, and apically to the implant body.

Under a 30° angled load originating from the distal direction with 200 N, the highest averaged normalized strain values for the BL implant were mesial to the implant body at the level of the lower third of the implant and distal to the implant body in the crestal region.

For the BLT implant, maximum strain values were observed in the crestal and middle thirds of the implant body, as well as distal to the implant body in the crestal region.

In the case of the BLX 3.75 implant, maximum strain values were found mesial to the implant body in the crestal and middle regions relative to the implant body, and distal to the implant in the crestal region.

With the BLX 4.5 implant, maximum strain values were measured mesial, centrally, and crestally to the implant body.

**FIGURE 9:**
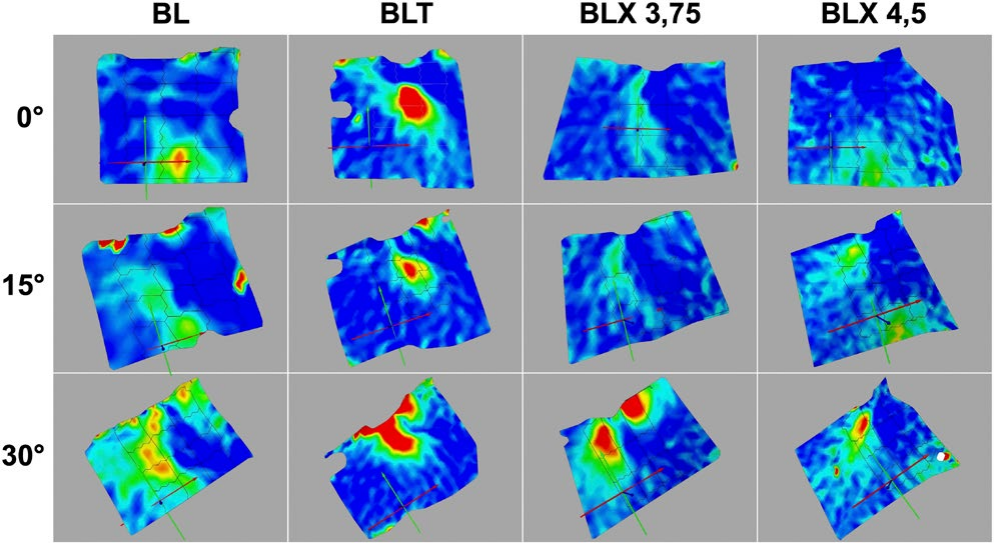
Representation of ARAMIS PROFESSIONAL Software illustrating three distinct loading conditions across four different implant types.

Figure 9 illustrates the strain pattern on the bone surface for the four different implant types under three loading directions (axial 0°, non‐axial 15°, and non‐axial 30°). The representation in ARAMIS PROFESSIONAL software as a heatmap depicts the intensity of strain, with colors indicating the gradual transition from low (blue) to high strain (red).

## 4 Discussion

To our knowledge, there are no studies that have investigated the influence of implant macrodesign on the strain behavior in surrounding bone using DIC. However, there are numerous reviews based on finite element analysis addressing this topic [26, 38, 39]. Therefore, it is important to compare the findings from studies utilizing finite element analysis with the newly presented results.

In the finite element analysis study by Holmgren et al. [22], it was reported that angled loads lead to higher stress values. Consistent with this finding, our study observed similar trends, except for the BLT implant and the BLX 4.5 implant when comparing axial loading to non‐axial loading (15°), where no increase in strain or even a slight decrease was noted. This suggests that the conical design of the BLT implant and the BLX 4.5 implant, along with their larger diameter, may allow for a more stable response to non‐axial loads. The BLX 3.75 implant, although also conical, showed a moderate increase by a factor of 1.14. These observations indicate that BLT and BLX 4.5 implants may exhibit less sensitivity to non‐axial loads compared with parallel‐walled implants and implants with smaller diameters.

When changing the direction of load from 15° to 30°, all implant types showed an increase in strain. Similarly, when comparing axial loading to non‐axial loading at a 30° angle, all implants also exhibited higher strain values.

Furthermore, Holmgren et al. [22] concluded that the maximum stress under axial loading does not significantly vary between different diameters. Consistent with their findings, our study also observed similar results, as larger diameters did not lead to lower strain values.

According to Holmgren et al. [22], it was also observed that under non‐axial loading, a larger diameter resulted in a lesser increase in stress. This statement contrasts with the results presented here, as both the increase in strain between axial loading and non‐axial loading at a 30° angle for the BLT implant (increase in average strain values by a factor of 1.49) and the BLX 3.75 implant (factor of 1.82) were lower compared with the BLX 4.5 implant (factor of 2.02).

Chun et al. [40] reported that under the same force and a non‐axial load of 15°, the maximum stress doubles compared with axial loading. In our study, we observed only a 1.67‐fold increase in the case of the BL implant, which may be attributed to differences in measurement methods, as the results are generally similar. For the BLT, BLX 3.75, and BLX 4.5 implant types, there was no significant increase in strain, and in fact, the BLT and BLX 4.5 implants showed a slight decrease in maximum averaged strain values. One possible explanation for this could be the implant shape, as the BL implant is the only one with a parallel‐walled structure, whereas the others are conically shaped.

Several studies have already demonstrated that conical implants generate higher stresses than cylindrical implants of comparable dimensions. Specifically, stress peaks have been observed, particularly in the crestal region [27, 41, 42]. In the present study, we were able to confirm this statement under axial loading, as the parallel‐walled BL implant exhibited the lowest strain values. In contrast, the conical BLT implant showed significantly higher strain values. Both the BL and BLT implants had the same length and diameter at the implant neck, ruling out these design features as causes for the differences in strain values. Thus, we cannot agree with the statements of Huang et al. [28] and Paracchini et al. [43] that conical implants reduce stresses in bone under axial loading. Under non‐axial loading (30°), a heterogeneous picture emerges. The conical BLT implant exhibits higher strain values, while the two conical BLX implants show lower strain values compared with the parallel‐walled BL implant. This variability may be related to the cone angle and thread depth of the implant types. The higher cone angle of the BLT implant could lead to greater stress concentration and thus higher strain values. In contrast, the lower cone angles and greater thread depth of the BLX implants may result in better load distribution and lower strain values under non‐axial loading.

Upon inspection of crestal bone strain comparing different implants under axial loading and non‐axial loading at 15°, the parallel‐walled BL implant exhibits lower averaged normalized strain values in areas a, b, and c than the other three conical implants. Under non‐axial 30° loading, only the BLT and BLX 3.75 implants show higher averaged normalized strain values in the crestal area, whereas the BLX 4.5 implant has lower averaged normalized strain values.

Petrie and Williams [27] also reported that implant diameter has a greater impact on reducing crestal bone strain than implant length or taper. According to Petrie, increasing the diameter of the implant led to up to a 3.5‐fold reduction in strain, while an increase in length resulted in a 1.65‐fold decrease. Conversely, taper increased bone strain, leading to a 1.65‐fold increase.

These findings contrast with the results of the present study. The BLX 4.5 implant exhibited higher strain values under non‐axial loading (30°) compared with the BLX 3.75 implant with a smaller diameter. These maximum strains were measured on the mesial side of both implants. For the BLX 3.75 implant, the maximum strain was in zone a, the mesial crestal area, whereas for the BLX 4.5 implant, maximum strain values were in zone d, mesial to the implant at the level of the middle third. Additionally, our study observed that with increasing implant length under non‐axial loading (30°), strain decreased. The BLX 3.75 implant, 14 mm in length, showed the lowest strains, followed by the 12 mm BLX 4.5 implant. The highest strains were observed in the 10 mm BL and BLT implants. This observation suggests that the larger contact area of longer implants with the bone leads to a more even distribution of load, thereby reducing local bone strain. It is important to note that the comparability of strain values may be limited not only by variation in implant length but also by differences in diameter, taper, and thread geometry.

Oliveira et al. [10] conducted a finite element analysis to study bone strains under a 150 N load applied at a 30° angle from the distal direction into the implants. They found that strain concentrated contralaterally to the force direction relative to the implant. This finding aligns with observations in our study for the majority of implants. The BL, BLX 3.75, and BLX 4.5 implants exhibited highest strains contralateral to the implant under 200 N and 30° angled loading. Only the BLT implant showed maximal strains ipsilaterally. One possible explanation for this phenomenon could be the microthreads on the BLX implants. Microthreads may lead to a stronger concentration of stress on the contralateral side, thereby altering stress distribution. This altered stress distribution due to microthreads could explain why BLX implants show lower strains under non‐axial loading. Additionally, the BL implant, despite lacking microthreads, also showed higher strain on the contralateral side, suggesting that factors beyond microthreads, such as overall thread design and implant structure, may play a role. This observation suggests new avenues for further studies examining the role of microthreads and other structural features of implants in stress distribution and bone strain.

The presented results are subject to several limitations. First, while porcine mandibles were chosen for their anatomical similarities to human bone, they cannot perfectly replicate the complex biological and structural properties of the human jaw. Second, although the periosteum was removed to facilitate the application of the stochastic pattern, the remaining rough and irregular bone surface may have influenced the accuracy of strain measurements. Third, the in vitro experimental setting, which lacks the dynamic biological environment, healing processes, and soft‐tissue support found in the human oral cavity, may not fully represent clinical conditions. As such, the direct translation of these findings to clinical practice should be approached with caution. Future research using human specimens, in vivo studies, or refined in vitro models that more closely mimic clinical realities would further enhance the relevance and applicability of the results.

From a clinical perspective, these findings underscore the importance of selecting an implant geometry that aligns with the expected loading scenario. For instance, when predominantly axial loads are anticipated, implants with parallel‐walled designs may reduce peak bone strain, potentially enhancing long‐term stability. In contrast, under non‐axial loading conditions, certain conical implants—especially those with optimized taper angles and microthread configurations—could better distribute forces, minimizing localized strain concentrations. This insight can guide clinicians in choosing implant designs tailored to individual patient anatomies and load conditions, ultimately improving clinical outcomes and reducing the risk of implant complications.

Given the measured surface changes, a direct comparison between finite element analysis and DIC as measurement methods would be highly interesting and a potential future study approach. This could directly verify surface strains and relate them to simulations of internal processes. To better differentiate the influence of various design parameters on peri‐implant strain behavior, future studies could benefit from using implants differing in a single characteristic. For instance, implants with identical diameter and taper but varying lengths could be a useful example for such investigations.

## 5 Conclusions

Under axial loading, parallel‐walled implants (BL) exhibited lower strain values on the surface of peri‐implant bone compared with conical implants. However, when subjected to non‐axial loading, they showed the greatest increase in strain (BL with an increase by a factor of 2.96). Under 30° non‐axial loading, long, conical implants with smaller diameters (BLX 3.75) resulted in lower strains in the peri‐implant bone compared with implants with larger diameters and shorter lengths. Short, conical implants (BLT) showed higher strain values, but overall lower relative increases (factor of 1.49) compared between axial and non‐axial loading.

It was observed that non‐axial loading led to higher strains than axial loading. Specifically, under non‐axial loading, higher strains were predominantly observed on the side opposite to the direction of applied force. Based on our investigations, microthreads in BLX implants were found to potentially lead to increased stress concentration on the contralateral side.

To build upon these findings, future research should aim to conduct in vivo experiments or employ more clinically realistic models to better understand how variations in implant geometry interact with the complex biological environment. Such studies could further validate our results, refine implant design guidelines, and ultimately improve long‐term clinical outcomes by informing evidence‐based implant selection and placement strategies.

## Author Contributions

Conceptualization: R.E.M. and C.M. Methodology: M.L. Software: R.E.M. and C.M. Validation: R.E.M. and C.M. Formal analysis: R.E.M. Investigation: M.L. Resources: M.W. Data curation: R.E.M. and M.L. Writing – original draft preparation: R.E.M. and M.L. Writing – review and editing: C.M. and M.W. Visualization: M.L. Supervision: R.E.M. Project administration: R.E.M. Funding acquisition: R.E.M. All authors have read and agreed to the published version of the manuscript.

## Conflicts of Interest

The authors declare no conflicts of interest.

## Data Availability

The data that support the findings of this study are available from the corresponding author upon reasonable request.
